# Review of clinical studies of perampanel in adolescent patients

**DOI:** 10.1002/brb3.505

**Published:** 2016-06-28

**Authors:** Heung Dong Kim, Ching‐Shiang Chi, Tayard Desudchit, Marina Nikanorova, Anannit Visudtibhan, Charcrin Nabangchang, Derrick W. S. Chan, Choong Yi Fong, Kai‐Ping Chang, Shang‐Yeong Kwan, Fe De Los Reyes, Chao‐Ching Huang, Surachai Likasitwattanakul, Wang‐Tso Lee, Ada Yung, Amitabh Dash

**Affiliations:** ^1^ Division of Pediatric NeurologyYonsei University Severance Children's HospitalSeoulKorea; ^2^ Department of PediatricsTungs' Taichung Metro Harbor HospitalTaichungTaiwan; ^3^ Department of Paediatric NeurologyKing Chulalongkorn Memorial HospitalBangkokThailand; ^4^ Children's DepartmentDanish Epilepsy Centre FiladelfiaDianalundDenmark; ^5^ Division of NeurologyDepartment of PediatricsFaculty of Medicine Ramathibodi HospitalMahidol UniversityBangkokThailand; ^6^ Department of PaediatricsPhramongkutklao HospitalBangkokThailand; ^7^ Department of PaediatricsKK Women's and Children's HospitalSingapore CitySingapore; ^8^ Division of Paediatric NeurologyDepartment of PaediatricsFaculty of MedicineUniversity of MalayaKuala LumpurMalaysia; ^9^ Department of PediatricsTaipei Veterans General HospitalTaipeiTaiwan; ^10^ Department of NeurologyTaipei Veterans General HospitalTaipeiTaiwan; ^11^ Department of PediatricsMother Seton HospitalCamarines SurPhilippines; ^12^ Department of PediatricsCollege of MedicineTaipei Medical UniversityTaipeiTaiwan; ^13^ Department of PediatricsFaculty of MedicineSiriraj HospitalMahidol UniversityBangkokThailand; ^14^ Department of PediatricsNational Taiwan University HospitalTaipeiTaiwan; ^15^ Department of PediatricsThe Duchess of Kent Children's HospitalSandy BayHong Kong; ^16^ Eisai Pharmaceuticals India Pvt., Ltd.MumbaiIndia

**Keywords:** Adolescent, Anticonvulsants, consensus, epilepsy, perampanel, receptors AMPA

## Abstract

**Aim:**

To assess the clinical trial and real‐world data for adjunctive perampanel in adolescents and develop consensus recommendations to guide the use of perampanel in this population in clinical practice.

**Methods:**

In May 2015, 15 epilepsy experts attended a Consensus Development Meeting to assess the clinical trial data for perampanel, specific to the adolescent age group (12‐17 years) and develop consensus treatment recommendations.

**Results and Discussion:**

Analysis of the adolescent subgroup data of three pivotal placebo‐controlled, double‐blind, phase 3 trials investigating perampanel in patients with ongoing focal epileptic seizures despite receiving one to three antiepileptic drugs found that perampanel 4–12 mg was superior to placebo. The tolerability profile of perampanel was generally acceptable. Adolescent patients receiving long‐term treatment with perampanel in an open‐label extension study maintained improvements in seizure control compared with baseline, with a favorable risk‐benefit profile. A phase 2 study showed that perampanel had no clinically important effects on cognitive function, growth, and development.

**Conclusion:**

Perampanel is a welcome addition to the armamentarium of existing antiepileptic drugs as it represents a new approach in the management of epilepsy, with a novel mechanism of action, and the potential to have a considerable impact on the treatment of adolescents with epilepsy.

## Introduction

Glutamate is the mediator of most fast excitatory neurotransmission in the central nervous system. There are three classes of ionotropic receptors, each with distinct physiological roles that mediate glutamate's fast excitatory neurotransmission at the synapses: *α*‐amino‐3‐hydroxy‐5‐methyl‐4‐isoxazolepropionic acid (AMPA), N‐methyl‐d‐aspartate (NMDA), and kainate receptors (Rogawski 2011). AMPA receptors are distributed throughout the brain and they are particularly prominently expressed in areas relevant to epilepsy, including the hippocampus and amygdala. These receptors form an integral part of every network in the brain that requires synaptic transmission by glutamate. The AMPA receptors play a fundamental role in fast excitatory synaptic transmission, and therefore are considered to be more relevant as a therapeutic target than NMDA and kainate receptors (Rogawski 2011). Perampanel (Fycompa^®^, Eisai Co., Ltd, Tokyo, Japan) was developed specifically to target AMPA receptors. Perampanel is a potent, orally active, noncompetitive, highly selective ionotropic AMPA glutamate receptor antagonist indicated for the treatment of partial‐onset seizures with or without secondarily generalized seizures (Hanada et al. 2011) in patients with epilepsy aged 12 years and older. Perampanel is approved in more than 45 countries, including the USA and in the EU, for adjunctive treatment of partial seizures with or without secondarily generalized seizures in patients with epilepsy aged ≥12 years of age.

## Methods

In May 2015, a panel of epilepsy experts from Asia met in Taipei, Taiwan, to discuss the clinical trial data for perampanel specific to the adolescent age group (12‒17 years). The objectives of this panel were to develop consensus treatment recommendations for perampanel use in adolescent patients based on evidence from the published literature, clinical trial experience, and practical experience in routine clinical practice, and to provide clinical recommendations for use in real‐world settings. The recommendations made are based on the published literature and clinical trial, and real‐world experience; consensus was reached after discussion within the group of epilepsy experts. The aim of the report is to provide an up‐to‐date overview of clinical trial data for perampanel in adolescents, including treatment recommendations, data gaps, and future directions, to guide the use of perampanel in the adolescent population with epilepsy.

## Discussion

### Mechanism of action of perampanel

Perampanel selectively inhibits AMPA‐induced calcium influx, thus reducing neuronal excitation (Rogawski 2011). Although perampanel is highly selective for AMPA receptors, it also has a weak effect on kainate receptors, but has not been found to interact with other molecular targets, including NMDA receptors, at relevant concentrations. In cultured rat cortical neurons, perampanel acts in a concentration‐dependent manner, with a 50% inhibitory concentration of 93 nM compared with 12.5 *μ*M for the noncompetitive AMPA receptor antagonist GYKI52466 (Hanada et al. 2011). AMPA receptor antagonists have a broad spectrum of anticonvulsant activity, being effective against fully kindled seizures (Rogawski 2011).

### Randomized clinical trials

**Figure 1:**
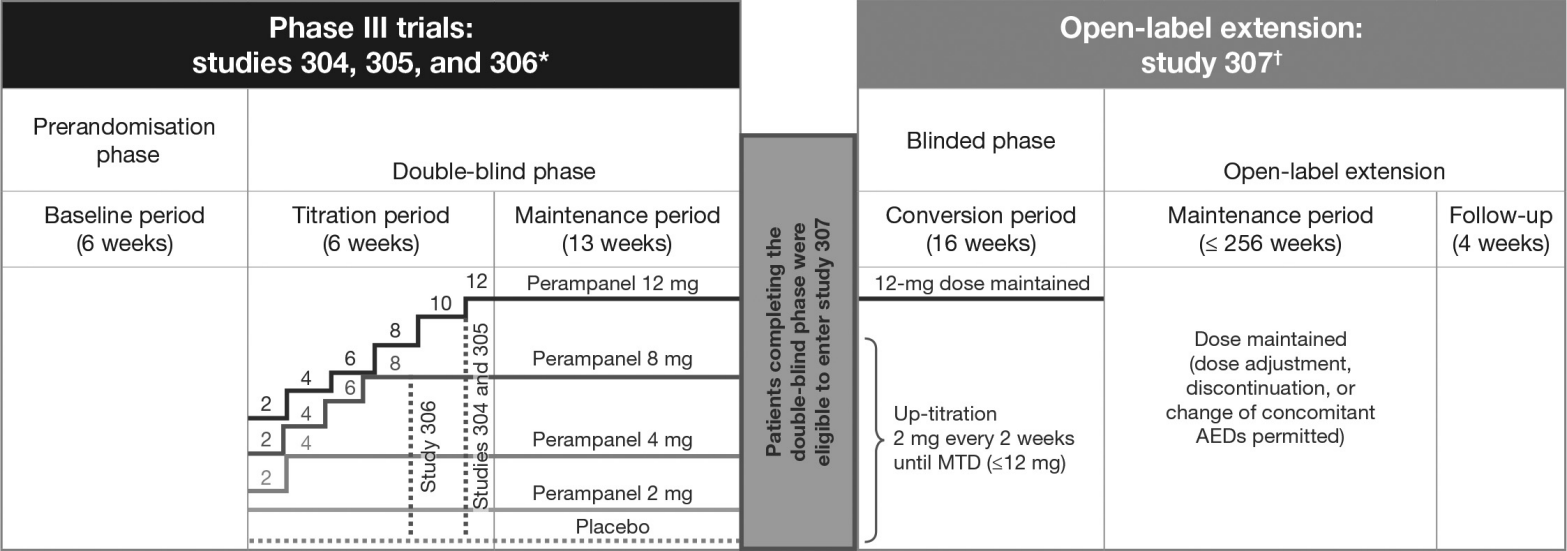
Design overview of three randomized controlled phase 3 trials and an open‐label extension study of perampanel. AED, antiepileptic drug; MTD, maximum tolerated dose. *Studies 304 (French et al. 2012), 305 (French et al. 2013), and 306 (Krauss et al. 2012). ^†^Study 307 (Krauss et al. 2013).

**Figure 2:**
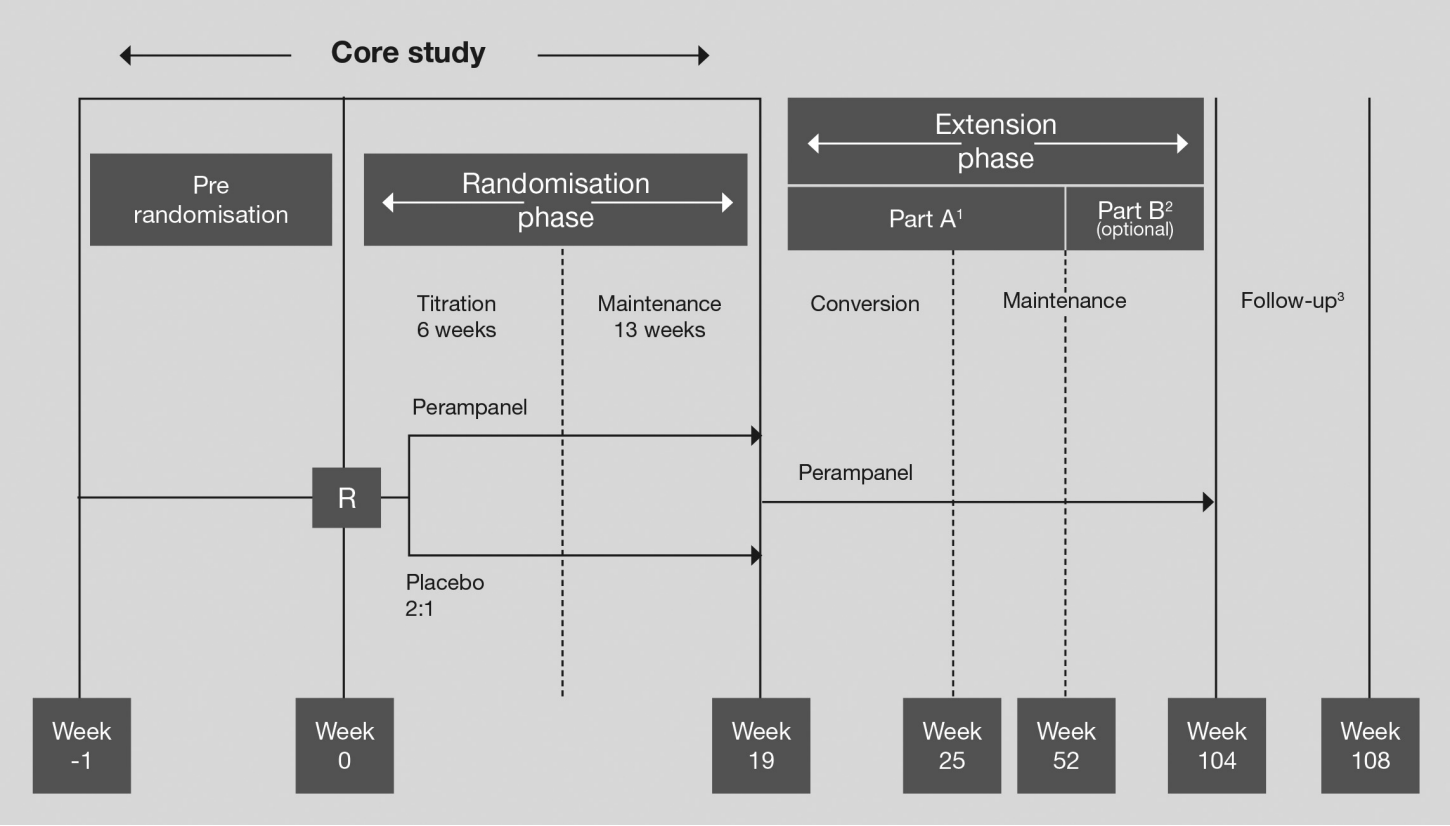
Design of a phase 2 study of perampanel (study 235) (Hussein et al. 2015; Pina‐Garza et al. 2015; Renfroe et al. 2015). R, randomization. ^1^All patients were retained to the last visit of extension part A. ^2^Part B was optional (a patient proceeded to or completed part B if perampanel was not commercially available or extended‐access program 401 was not in place in their country of residence). ^3^Follow‐up was conducted for all patients 4 weeks after their last on‐treatment visit.

Three multinational, multicenter, double‐blind, randomized, placebo‐controlled, phase 3 trials (studies 304 [NCT00699972] (French et al. 2012), 305 [NCT00699582] (French et al. 2013) and 306 [NCT00700310] (Krauss et al. 2012); Fig. 1), comprising a study population of adults and adolescents (age 12–17 years) were done to establish the minimum effective dose and the dose range (2–12 mg) of once‐daily perampanel. Patients with refractory epilepsy who were taking one to three approved antiepileptic drugs (AEDs) at baseline, but were still having uncontrolled partial‐onset seizures were enrolled. Study 306 assessed the low to middle dose range (2, 4, and 8 mg) (Krauss et al. 2012). The two other trials, studies 304 and 305, had identical methodology and assessed the higher daily doses of 8 and 12 mg (French et al. 2012, 2013). Study 307 (NCT00735397) was an open‐label extension study of patients completing the double‐blind phase of the three pivotal phase 3 trials (Krauss et al. 2013). Another recent placebo‐controlled phase 2 study was designed to determine the effect of perampanel on cognition, growth, safety, tolerability, and pharmacokinetics (PK) in adolescents (study 235; NCT01161524; Fig. 2; Hussein et al. 2015; Pina‐Garza et al. 2015; Renfroe et al. 2015). An observational retrospective multicenter survey provided real‐world clinical data on the effectiveness and tolerability of perampanel in children and adolescents (age 2–17 years) with refractory epilepsy (Biró et al. 2015).

#### Consensus statement 1

Perampanel has a novel mechanism of action. AMPA receptors are of primary importance based on a rational hypothesis of seizure initiation and spread. This novel mechanism of action can be considered as a rational combination therapy in patients with partial‐onset seizures who have failed to gain control with other AEDs.

### Efficacy and safety of perampanel in adolescents

In a pooled analysis of the three randomized, controlled, phase 3 trials (studies 304, 305, and 306) (French et al. 2012, 2013; Krauss et al. 2012), primary efficacy endpoints were median percentage change in frequency of all partial seizures per 28 days (baseline vs. double‐blind phase) and percentage of patients achieving *a* ≥ 50% reduction in the frequency of all partial seizures per 28 days (50% responder rate; baseline vs. maintenance phase). The median percentage changes in the frequencies of complex partial seizures plus secondarily generalized seizures and secondarily generalized seizures only were assessed as secondary and exploratory endpoints, respectively (Steinhoff et al. 2013).

A total of 1480 patients were enrolled in studies 304, 305, and 306 (French et al. 2012, 2013; Krauss et al. 2012). Of these, 145 adolescent patients were randomized to adjunctive therapy with either perampanel (*n *=* *100) or placebo (*n *=* *45); 143 received ≥1 dose of study drug and were included in the analysis. In the three studies, 79% of patients receiving perampanel and 89% of those receiving placebo were taking two or three baseline AEDs.

**Figure 3:**
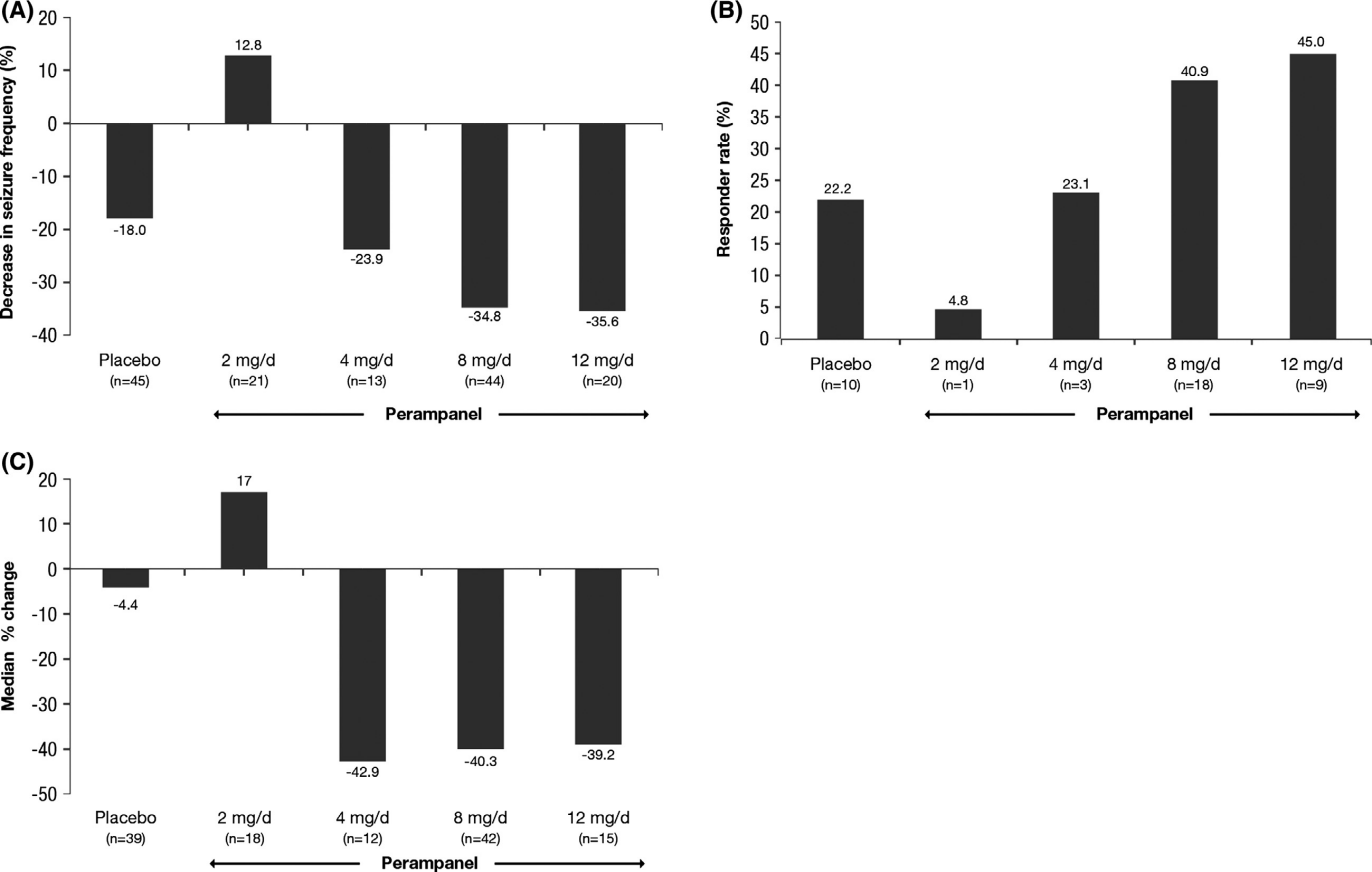
Pooled efficacy data from pivotal phase 3 studies 304 (French et al. 2012), 305 (French et al. 2013), and 306 (Krauss et al. 2012). (A) Median percentage change in seizure frequency per 28 days of treatment versus baseline; (B) 50% responder rates; and (C) median percentage change for complex partial seizures plus secondarily generalized seizures (Steinhoff et al. 2013). The subanalysis was not powered for statistical analysis.

The pooled data from the three trials show that, in perampanel‐treated adolescents, efficacy outcomes for the adolescent age group (12–17 years) were consistent with the overall findings of the phase 3 studies (French et al. 2012, 2013; Krauss et al. 2012). Seizure frequency and responder rate data supported an effective dose range of perampanel, 4–12 mg in adolescent patients, providing seizure reduction in patients with refractory partial‐onset seizures (Fig. 3).

Among adolescents, the PK profile of perampanel was consistent across age groups and did not vary by any demographics. The predicted probability of response increased with perampanel exposure. Between baseline and end of treatment, perampanel was not associated with any clinically significant changes in mean hematology and clinical chemistry values, vital signs, mean electrocardiogram parameters or skin photosensitivity (Rosenfeld et al. 2015). Perampanel demonstrated a favorable risk–‐benefit profile. The most common (observed in ≥5% of patients) treatment‐emergent adverse events (TEAEs) were dizziness, somnolence, aggression, decreased appetite, and rhinitis (Rosenfeld et al. 2015). Of note was the incidence of aggression, which was higher in patients treated with perampanel (*n *=* *8) than with placebo (*n *=* *0). The incidence of aggression in adolescents was also higher than that observed in adults aged 18–65 years (8% vs. 1%). Of the eight adolescents who experienced aggression, two experienced serious aggression‐related AEs (both male patients: one receiving 2 mg and the other receiving 12 mg) and four required drug interruption or adjustment; the patient randomized to the 12 mg group discontinued treatment. The AEs were described as aggressive behavior, temper tantrums, behavioral aggression, or increased aggressive behavior.

#### Consensus statement 2

Adolescent subgroups display similar outcome tendencies to the overall study group (no significant differences between the adult and adolescent groups). Adjunctive perampanel treatment in adolescents produced better seizure control than placebo and sustained seizure frequency improvements for up to 2 years of follow‐up. Patients with secondarily generalized seizures achieved greater seizure freedom. Adjunctive perampanel in this group had a generally favorable tolerability profile. There could be a slight increase in aggression in the adolescent patient group.

In the observational real‐world study of 58 patients (mean age, 10.5 years; range, 2–17 years) with various refractory epilepsies, 18 patients achieved ≥50% seizure reduction for a response rate of 31% after 3 months (Biró et al. 2015). Five patients (9%) achieved complete seizure control and five patients (9%) experienced aggravation of seizures. The most frequent AEs were reduced vigilance or fatigue (28%) and behavioral changes (24%).

#### Long‐term results

In the open‐label extension study 307, long‐term safety and tolerability of perampanel as an adjunctive treatment for refractory partial‐onset seizures was evaluated. In addition, the maintenance effect of perampanel for treatment of refractory partial‐onset seizures was assessed (Krauss et al. 2013). Of 129 adolescent patients completing the pivotal phase 3 studies, 124 enrolled in the extension study; 122 patients were included in the intention‐to‐treat analysis set and 121 in the safety analysis set. In this study, 82% of patients in the safety analysis set were taking two or three AEDs.

**Figure 4:**
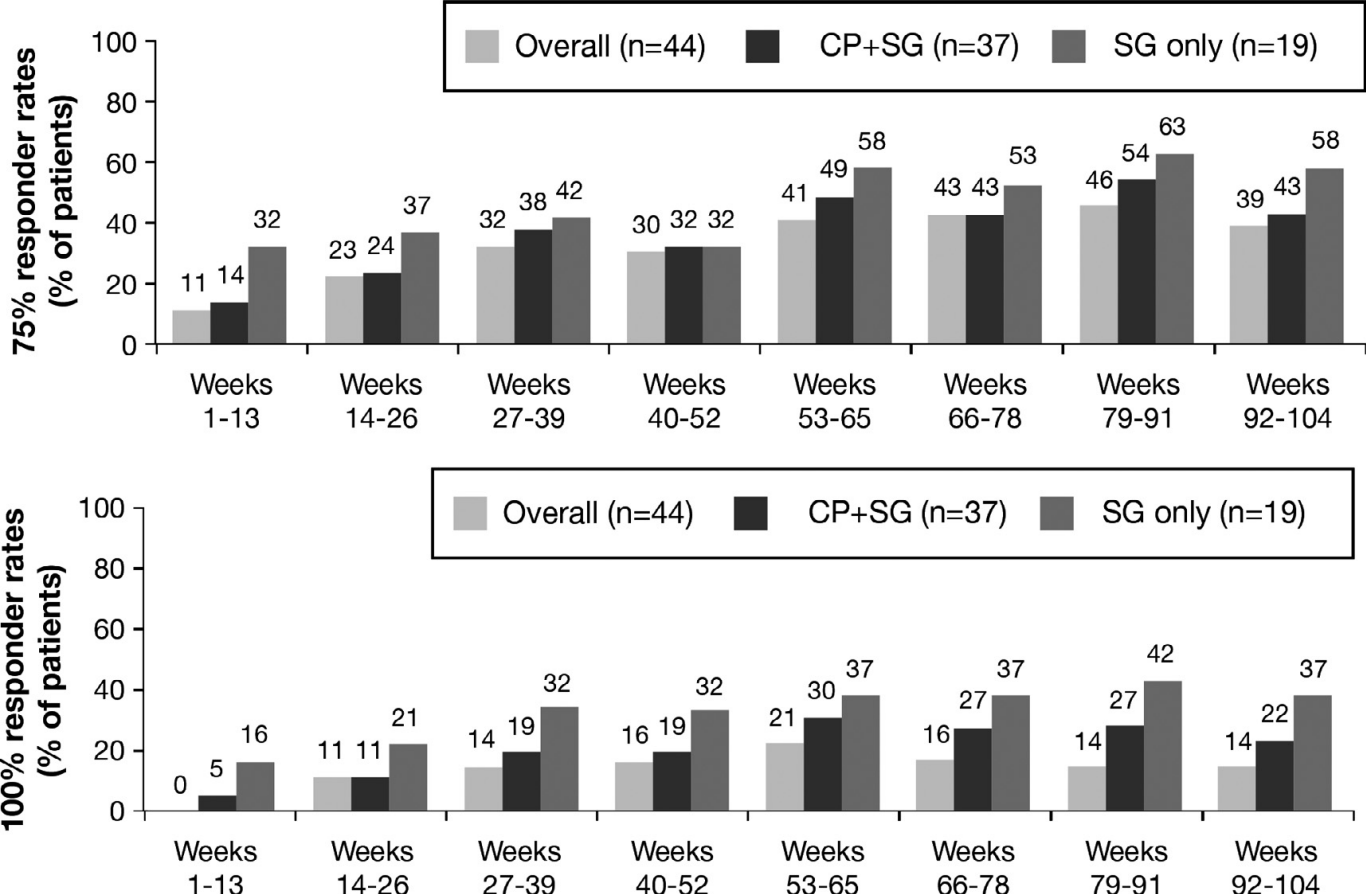
Responder rates in the open‐label extension study 307 (Krauss et al. 2013; Rosenfeld et al. 2015). CP, complex partial; SG, secondarily generalized.

Interim results from this trial showed that, adolescent patients receiving long‐term treatment with perampanel maintained improvements in seizure control compared with baseline. The decrease in seizure frequency was consistent and maintained in those patients over at least 1 year of perampanel exposure (Fig. 4) (Renfroe et al. 2014). Consistent with the pivotal phase 3 trials (French et al. 2012, 2013; Krauss et al. 2012), perampanel had a favorable tolerability profile in adolescent patients with refractory partial‐onset seizures over the longer term. The most common treatment‐related TEAEs requiring perampanel interruption or dose adjustment were dizziness (13.2%, n=16), somnolence (11.6%, n=14), aggression (6.6%, n=8), irritability (2.5%, n=3), asthenia, ataxia, convulsion,and abnormal behaviour(n=2;1.7% for each) (Steinhoff et al. 2013). The discontinuation rate due to TEAEs was 14.9% (n=18) and the rate of serious AEs in extension study was 14.0% (n=17). Behavioral TEAEs noted during the extension study included aggression (18.2% n=22), insomnia (6.6% n=8), abnormal behavior (4.1% n=6), anxiety (4.1% n=5), and anger (3.3% n=4). Of the 22 patients experiencing aggression, 21 were receiving higher dose of perampanel, 8–12 mg. A higher incidence of aggression was observed among adolescents compared with adults. However, most cases were mild or moderate (mild [*n *=* *9], moderate [*n *=* *10], severe [*n *=* *3]); three patients with aggression discontinued the study (Steinhoff et al. 2013). Adolescent patients treated with perampanel and their caregivers need to be aware of the potential for aggressive behavior, especially during titration (Rosenfeld et al. 2015). If aggression is noted, a trial of alternate day dosing could be considered (Marina Nikanorova, Danish Epilepsy Centre Filadelfia, pers. Comm. 2015). Overall, perampanel demonstrated a favorable risk–‐benefit profile.

#### Consensus statement 3

Patients receiving perampanel should be monitored for AEs related to irritability and aggression, particularly during dose titration and at higher doses. Patients and caregivers should be counseled regarding the potential risk of psychiatric or behavioral AEs. Any prior history of psychiatric or behavioral disorders, family history of psychiatric disorders, or history of aggression with prior AEDs should be noted, and slower dose titration and closer monitoring could be considered. There is no clear mechanism or hypothesis for aggression. Young adults are in the process of development, and hence could be more sensitive to drug‐induced aggressive behavior.

### Effect of perampanel on cognition, growth, and development

Study 235 was conducted to compare the short‐term effect on cognition of adjuvant perampanel versus placebo in 133 adolescents with inadequately controlled partial‐onset seizures using the Cognitive Drug Research (CDR) System Global Cognition Score (Hussein et al. 2015; Pina‐Garza et al. 2015; Renfroe et al. 2015; Meador et al. 2016). The primary outcome measure was change from baseline (week 0) to end of maintenance therapy (week 19) in global cognition score. Key secondary outcome measures were change from baseline in five CDR System cognitive domains of: power of attention; continuity of attention; quality of episodic memory; quality of working memory; and speed of memory.

Data from this trial showed that adjunctive therapy with perampanel up to 12 mg/day was associated with improved seizure control and was well tolerated in adolescents with inadequately controlled partial seizures (Renfroe et al. 2015). Patients who received perampanel reported a higher incidence of aggression and irritability compared with placebo in this study. However, these findings are consistent with adolescent data from the phase 3 studies (French et al. 2012, 2013; Krauss et al. 2012).

#### Consensus statement 4

The once‐daily night‐time dosing, simple titration schedule, and long half‐life may offer ease of use and potential for adherence in the adolescent group. The advantages of once‐daily night‐time dosing include the potential for a more stable mean drug concentration over time, improved tolerability profile, maximal use of the therapeutic window, and the possibility to achieve better seizure control. Improvements in overall treatment effectiveness may therefore increase adherence in adolescent patients and the long half‐life may offer additional protection against lack of adherence in case of a missed dose. Careful and slow titration over 2–4 weeks in adolescent patients is recommended.

Mean change in CDR System Global Cognition Score from baseline showed that perampanel did not significantly influence cognitive characteristics (*P *=* *0.145; Table 1; Meador et al. 2016). No significant differences were observed with change from baseline in Power of Attention (*P *=* *0.219) and Quality of Working Memory (*P *=* *0.579). There were small, but significant, differences in favor of placebo for Continuity of Attention (*P *=* *0.013) and Speed of Memory (*P *=* *0.032), while Quality of Episodic Memory (*P *=* *0.012) was improved in patients receiving perampanel (Table 1).

**Table 1:** Effect of perampanel on cognitive function assessed by CDR System Global Cognition Score in study 235 – full analysis (Meador et al. 2016)

Parameter	LS mean change (SE)		Difference in LS means (95% CI)	*P*‐value
	Placebo (*n* = 44)	Perampanel (*n* = 79)	Perampanel versus placebo	
CDR System Global Cognition Score	1.6 (1.3)	−0.6 (1.0)	−2.2 (−5.2 to 0.8)	0.145
Power of attention	−2.7 (3.0)	−6.9 (2.3)	−4.2 (−11.0 to 2.6)	0.219
Quality of working memory	2.0 (1.5)	1.1 (1.2)	−1.0 (−4.4 to 2.5)	0.579
Continuity of attention	1.6 (1.2)	−1.7 (0.9)	−3.3 (−6.0 to −0.7)	0.013
Quality of episodic memory	−1.2 (1.5)	3.0 (1.1)	4.2 (0.9 to 7.5)	0.012
Speed of memory	7.0 (2.7)	0.3 (2.1)	−6.6 (−12.7 to −0.6)	0.032

SE, standard error; CI, confidence interval; LS, least squares.

Statistical significance – (*P* < 0.05).

The most commonly reported TEAEs for perampanel‐treated patients were dizziness and somnolence. Aggression was reported in 2.1% of placebo‐treated patients and 8.2% of perampanel‐treated patients. Three of seven perampanel‐treated patients with aggression required dose modification and two had serious aggression, although none required treatment discontinuation.

Perampanel steady state exposure studies indicated no effect of exposure to perampanel on CDR System Global Cognition Score, Quality of Working Memory, and Speed of Memory (Hussein et al. 2015). PK–pharmacodynamic (PD) relationships were apparent for Power of Attention (beneficial), Continuity of Attention (worsening), and Quality of Episodic Memory (beneficial). These findings were further supported by PK–PD analyses using nonlinear mixed effects modeling.

#### Consensus statement 5

No negative effect of perampanel exposure on the primary study outcome measure of CDR System Global Cognition Score was observed. This substantiates the primary study endpoint results that there is no evidence of an overall short term effect of perampanel on cognitive function, as measured by CDR System Global Cognition Score, when compared with placebo.

**Figure 5:**
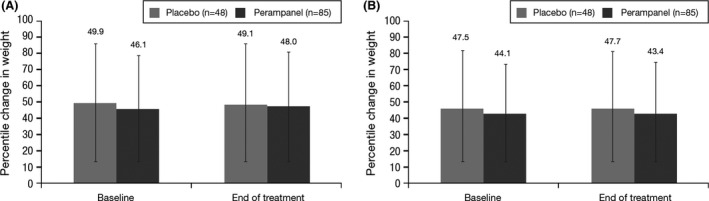
Percentile change from baseline in (A) weight and (B) height.

Overall, perampanel did not negatively impact growth and development compared with placebo (Pina‐Garza et al. 2015). Mean change in weight percentile decreased slightly for placebo (baseline (49.9%) to end of treatment (49.1%), −1.0; standard deviation [SD]: 4.9) and increased slightly for perampanel (baseline (46.1%) to end of treatment (48.0%), 1.9; SD: 6.7). Mean change in height percentile was similar for both the placebo (baseline (47.5%) to end of treatment (47.7%), −0.7; SD: 8.2) and perampanel groups (baseline [44.1%] to end of treatment [43.4%], −0.8; SD: 5.9; Fig. 5). The sex‐ and age‐specific percentiles for weight and height were calculated from the Centers for Disease Control and Prevention Growth Charts (Centers for Disease Control and Prevention). Insulin‐like growth factor‐1 (IGF‐1) decreased minimally with perampanel treatment (−1.1; SD: 113.9) and to a greater extent with placebo treatment (−13.9; SD: 93.9). There were minimal or no changes from baseline for thyrotropin, free thyroxin, and free triiodothyronine, with no difference between treatment groups. There were no clinically important changes in bone age from baseline to the end of treatment. When compared with placebo, perampanel did not negatively affect sexual development in either males or females (Tanner staging; Marshall and Tanner 1970).

#### Consensus statement 6

The average weight change observed in adolescents receiving perampanel is consistent with that expected for the general adolescent population and thus can be attributed to normal adolescent growth rather than a TEAE. Adjunctive perampanel therapy in adolescents with partial seizures showed no overall short‐term effects on growth and development relative to placebo. Long‐term effects of perampanel on cognition, growth and development in adolescents should be evaluated in future studies.

#### Consensus statement 7

All adolescent patients with refractory partial‐onset seizures should be reviewed by an epilepsy specialist when possible. Perampanel may be initiated by an epilepsy specialist, appropriately qualified pediatrician or general neurologist. Perampanel can be considered a second‐line adjunctive therapy option in patients aged 12 years and older with partial‐onset seizures. Perampanel may be combined with other AEDs with good efficacy outcomes. A higher dose of perampanel may be required in patients taking enzyme‐inducing AEDs.

#### Consensus statement 8

Perampanel should be initiated at a dose of 2 mg/day, taken at night, and titrated by increments of 2 mg every 2–4 weeks according to the clinical need to achieve the maximum tolerated dose (MTD; up to 12 mg/day). The administration schedule was established from the results of the clinical trials with fixed titration schedules but, based on real‐world clinical practice experience, slower titration rates are recommended for fewer side effects and better adherence in adolescent patients. Consider withdrawing perampanel if there is no evidence of clinical benefit once the MTD has been reached and maintained for an adequate period. Patients with partial‐onset seizures with motor symptoms or secondarily generalized seizures can have greater benefit with perampanel.

### Perampanel trial summary

Adjunctive therapy with perampanel (up to 12 mg/day) resulted in improved seizure control and was well tolerated in adolescents with inadequately controlled partial seizures. PK–PD results indicate that perampanel has no clinically important short term effects on overall cognitive function, growth and development in adolescents. Results for adolescents were comparable to the overall study population. However, aggression was more frequent in adolescents than in adults, in some cases leading to treatment discontinuation.

## Future directions

Several data gaps pertinent to perampanel were discussed, and many opportunities to define the future direction for optimizing the use of perampanel in the pediatric population and adolescents were presented by the meeting attendees. These suggestions are outlined below.

Reasons for the increased occurrence of aggression with perampanel treatment remain unclear, indicating a need to understand the underlying mechanism of treatment‐related aggression. This will help optimize treatment in this patient population. As such, the expert group suggested using postmarketing surveillance studies to begin understanding aggression in adolescent patients taking perampanel.

There was agreement that the safety and effectiveness of perampanel in pediatric patients younger than 12 years are required to inform drug use in younger patients. This could be facilitated with the implementation of open‐label studies in this population.

There are data supporting the use of perampanel in different types of epilepsy, including complex partial seizures and primary generalized tonic–clonic seizures (Steinhoff et al. 2014). Seizure freedom was achieved in 15% of patients and the responder rate was 50%. Nonetheless, robust data specific to adolescents are required as these are important for pediatricians in clinical practice.

Perampanel offers a potential benefit for the most refractory patients. Perampanel has low potential for drug interactions and predictable PK. Good tolerability is observed in most of the patients when assessing cognition, mood, and behavior. Long‐term effect of perampanel on cognition, growth and development in adolescents should be evaluated in the future studies.

Ease of use in a titration scheme and once‐daily formulation offer advantages over other AEDs. Perampanel may be combined with other AEDs with good efficacy outcomes. A higher dose of perampanel may be required in patients taking enzyme‐inducing AEDs. It was suggested to explore whether other AEDs can work synergistically with perampanel.

Presence of a comorbidity and quality of life are at least as important as seizure frequency in patients who are not seizure free. These data are required to recommend appropriate treatment for epilepsy in clinical practice.

Studies are required to evaluate perampanel as a monotherapy for the treatment of epilepsy. Perampanel should be evaluated from a pharmacoeconomic point of view.

In conclusion, perampanel is a welcome addition to the armamentarium of existing antiepileptic drugs as it represents a new approach in the management of epilepsy. Perampanel has a novel mechanism of action, and the potential to have a considerable impact on the treatment of adolescents with epilepsy. However, further research is needed to optimize perampanel therapy.

## Conflicts of Interest

All authors received an honorarium from Eisai Co., Ltd for their attendance at the expert meeting in Taipei, Taiwan, where this consensus was developed. Tayard Desudchit was an investigator on the 306, 307, and 235 studies. Marina Nikanorova and Anannit Visudtibhan have received speaker′s honoraria from Eisai Co., Ltd. Surachai Likasitwattanakul has received speaker's honoraria, funding for research and educational grants from Abbott, Eisai and GlaxoSmithKline. Amitabh Dash is an employee of Eisai Pharmaceuticals India Pvt., Ltd., Mumbai, India. Ching‐Shiang Chi, Charcrin Nabangchang, Derrick W. S. Chan, Choong Yi Fong, Kai‐Ping Chang, Heung Dong Kim, Shang‐Yeong Kwan, Fe De Los Reyes, Chao‐Ching Huang, Wang‐Tso Lee, and Ada Yung declare no other conflict of interest.
